# *Homalomena pineodora* essential oil nanoparticle inhibits diabetic wound pathogens

**DOI:** 10.1038/s41598-020-60364-0

**Published:** 2020-02-24

**Authors:** Nur Amiera Syuhada Rozman, Woei Yenn Tong, Chean Ring Leong, Mohd Razealy Anuar, Sabrina Karim, Siew Kooi Ong, Fahmi Asyadi Md Yusof, Wen-Nee Tan, Baharuddin Sulaiman, Mei Lee Ooi, Kok Chang Lee

**Affiliations:** 1 Drug Discovery and Delivery Research Laboratory, Universiti Kuala Lumpur, Malaysian Institute of Chemical and Engineering Technology, Lot 1988 Kawasan Perindustrian Bandar Vendor, Taboh Naning, 78000 Alor Gajah, Melaka Malaysia; 2 School of Distance Education, Universiti Sains Malaysia, 11800 Gelugor, Pulau Pinang Malaysia; 3 School of Biological Sciences, Universiti Sains Malaysia, 11800 Gelugor, Pulau Pinang Malaysia; 4 Faculty of Science, Universiti Tunku Abdul Rahman, Jalan Universiti, Bandar Barat (Perak Campus), 31900 Kampar Perak, Malaysia

**Keywords:** Drug delivery, Drug development

## Abstract

Essential oil of *Homalomena pineodora* inhibits diabetic pathogens; however, the activity was not sustainable when applied as wound dressing. This study aims to synthesise the essential oil nanoparticle using chitosan. The nanoparticles were synthesised with ion gelation method, confirmed by spectroscopic analysis. The spherical nanoparticles display a size of 70 nm, with strong surface charge of +24.10 mV. The nanoparticles showed an initial burst release followed by a slow release pattern for 72 h, following the first order of kinetic. The release behaviour was ideal for wound dressing. The antimicrobial activity was broad spectrum. The formation of nanoparticle enhanced the antimicrobial efficacy of the essential oil. The nanoparticle also showed a concentration-dependent killing behaviour on time–kill assay. In the 3D collagen wound models, the nanoparticles reduced the microbial growth by 60–80%. In conclusion, *H*. *pineodora* nanoparticles showed pharmaceutical potential in inhibiting microbial growth on diabetic ulcers.

## Introduction

Clinically, diabetic patients are at risk for developing foot ulcer, which ultimately leads to amputation. Amputation is a common surgical trauma among diabetic patients. Besides, wound healing in diabetic patients is frequently impaired where the delay in healing time is commonly associated with microbial infection^1^. Moreover, the presence of pathogens in the diabetic wound fluid prolongs the healing process^2^. Recently, various types of antimicrobial agent, nanoparticles and natural products have been incorporated into wound dressing. Unfortunately, the choice of antimicrobial agent provided no clear evidences if one was better than others in treating diabetic food ulcer because the data from previous reports were heterogeneous^2^. The inappropriate and indiscriminate use of antibiotics is a key reason contributing to antibiotic resistances in microorganisms. Silver finished textiles are very useful for wound care products. The incorporation of the heavy metals into a wound dressing, however, has been reported to cause skin allergy and inhibit fibroblast growth^3^.

*H*. *pineodora* is a novel plant species discovered in Peninsular Malaysia^4^. Several species of genus *Homalomena* have been used for traditional medicinal purposes^5^. In the previous study, we have reported that *H*. *pineodora* essential oil exhibited excellent bactericidal activity on diabetic wound pathogens^6^. However, the antimicrobial efficiency was not sustainable when applied as wound dressing. Thus, this study focused on the characterisation and antimicrobial efficiency of *H*. *pineodora* essential oil nanoparticles (EoNPs) using chitosan as encapsulant material.

There is an exceptional growth of research and applications in the area of nanoscience and technology, particularly in the field of medicine. Nanoparticles provide a colloidal drug delivery system that potentially eliminates the toxic chemical species absorbed on the surface of particle^7^. The nanoparticles are encapsulated by polymer, which release the drug by controlled diffusion from the core across the polymeric matrix. Hence, nanoparticles are widely used in the design of smart drug. The synthesized nanoparticles can be incorporated into wound dressing material, in order to improve the antimicrobial performance and sustain the antimicrobial activity^5^. The application of nanoparticles on wound healing has been reported, and most of these dressing gave a speedy recovery on the patients^8^.

## Experimental

### Synthesis of nanoparticles

*H*. *pineodora* leaves were collected from a plant nursery in the Universiti Sains Malaysia, Penang, Malaysia (Voucher specimens: No. 11748). Plant samples were collected using hand picking method. For 5 h, hydrodistillation was performed using a Clevenger-type apparatus with *n*-pentane as the collecting solvent^6^. The volatile essential oil was then placed at 4 °C in a sealed glass vial and protected from light before use.

The nanoparticles of essential oil have been synthesised with ion gelation method^9^. A total of 1% (w/v) of medium molecular weight chitosan (Bio Basic) was prepared in 1% acetic acid by agitating the solution overnight at 25 °C. Using laboratory centrifuge (Eppendorf centrifuge 5810 R), 100 ml of the solution was centrifuged for 1 h at 2550 *g*. The supernatant was then filtered through filters (Millipore) of 0.22 μm pore size. Then, to obtain a homogeneous mixture, 0.5% (w/v) of Tween 80 (Merck) was added as a surfactant and stirred for 2 h. The pH of solution with 1 M sodium hydroxide was adjusted to 5.3. To prepare the oil phase, at 11,000 rpm for 15 min (Silent Crusher M, Heidolph Instrument), 4 ml of 8% (w/v) of essential oil was dropped into the chitosan solution with homogenisation. Then, 40 ml of sodium polyphosphate (Merck), TPP 0.4% (w/v), was added drop by drop into the agitated emulsion at 25 °C. The agitation was continuously performed for 1 h in an ice bath. The formed particles were then collected for 1 h by centrifugation (Thermo Fisher Scientific) at 4 °C at 1700 *g*. The particles were washed thrice with deionised water, and the pellet was re-suspended in 60 ml distilled water. Then, the suspension was sonicated using Vibra-Cell Ultrasonic Processer (VCX 750, Sonics & Materials. Inc) equipped with a 13 mm probe for 15 min in ice at 60 rpm. The homogenised sample was stored at −80 °C and freeze-dried (Labconco). The particles were kept in desiccator until further analysis. For blank nanoparticles, the same step was repeated by replacing the essential oil with ethanol.

### Characterisation of nanoparticles

Microscopic observation was performed using transmission electron microscope (TEM) (Philips CM12) operating at 120 kV to observe the shape and sizes of nanoparticles. A Zetasizer (Nano-ZS90) version 7.11 instrument by Malvern was used to perform dynamic light scattering (DLS) particle size and surface charge of EoNPs. The size of nanoparticles was analysed using clear disposable cuvette adjusted to 2.0 mm measurement position and attenuator of 6. For zeta potential, DLS measurement was performed in a clear disposable zeta cell with the same instrument. The test temperature was set at 25 °C. The data were analysed using Zetasizer V2.2 software.

The infrared spectra of chitosan, *H*. *pineodora* essential oil, pure chitosan, EoNP and chitosan nanoparticles (ChNPs) were investigated by Fourier transform infrared (FTIR). FTIR spectroscopy was performed on an FTIR spectrometer (Thermo Scientific Nicolet IS10, USA). All spectra were recorded from 400 to 4000 cm^−1^ wavenumber range. BET surface area analysis was performed using a BET instrument (Micromeritics Accelerated SA and Porisimetry 2010 system) to measure the total surface area of EoNPs and ChNPs. The samples were degassed overnight to remove water and other contaminants at 110 °C. The data were analysed using 3Flex 4.04 software. XRD pattern was recorded using powder X-ray diffractometer (LabX XRD-6000, Shimadzu) using Cu Kα1 radiation. Samples were placed onto circular sample holder (16 mm diameter) and closed with the bottom plate. The X-ray source was operated at 40 kV and 40 mA. Step size was 0.017°. Diffraction intensity was measured in the reflection mode at a scanning rate of 2 °C/min for 2θ = 10–40° under continuous scan mode.

To determine the encapsulation efficacy and loading capacity, 10 mg of EoNPs was dispersed in 4 ml of 2 M hydrochloric acid in a universal bottle. Then, the sample was boiled at 95 °C in water bath. After that, 2 ml of ethanol was added into the sample. Then, 1 ml of sample was centrifuged at 9055 *g* (Thermofisher) for 10 min. After that, the absorbance of supernatant was measured using UV-VIS spectrophotometer at wavelength 325 nm. The blank sample also was prepared using same manner. The encapsulation efficiency and loading capacity of EoNP were calculated^10^.

1 $${\rm{Encapsulation}}\,{\rm{efficiency}}\,( \% )=\frac{{\rm{Drug}}\,{\rm{added}}-{\rm{Free}}\, \mbox{''}{\rm{untrapped}}\,{\rm{drug}}\mbox{''}}{{\rm{Drug}}\,{\rm{added}}}\times 100 \% $$

2 $${\rm{Loading}}\,{\rm{capacity}}\,( \% )=\frac{{\rm{Entrapped}}\,{\rm{drug}}}{{\rm{Nanoparticles}}\,{\rm{weight}}}\times 100 \% $$

The drug release study was performed to determine the Eo release behaviour from ChNP^9^. The freeze-dried EoNPs were immersed in artificial sweat solution (pH 5.5) with a ratio of 1:1000 (w/v). The sample was incubated at rotational speed of 60 rpm a37 °C. At regular time intervals of 0, 1, 2, 4, 8, 24, 48, and 120 h, 1 ml of sample was withdrawn and centrifuged at 18,894 *g* (Thermofisher). Then, the supernatant was extracted using hexane. The absorbance of sample was determined using UV-VIS spectrophotometer at wavelength 280 nm. The release of Eo was determined using calibration curve of essential oil (0.08–50.00 mg/ml).

## Antimicrobial Assays

The test bacteria used in this study include four Gram-positive bacteria [*Bacillus cereus*, *Bacillus subtilis*, *Staphylococcus aureus* and methicilin-resistant *Staphylococcus aureus*], eight Gram-negative bacteria [*Escherichia coli*, *Proteus mirabilis*, *Yersinia* sp., *Klebsiella pneumoniae*, *Shigella boydii*, *Salmonella typhimurium*, *Acinetobacter anitratus* and *Pseudomonas aeruginosa*] and two yeasts [*Candida albicans* and *Candida utilis*]. The clinical microbial strains were provided by the Hospital Seberang Jaya, Penang. The bacterial suspensions were prepared, and turbidity of the suspensions was adjusted according to 0.5 Mc Farland standard.

Disc diffusion assay was used to screen the antimicrobial efficacy of the EoNP on a wide spectrum of microorganisms^11^. A total of 100 μl of inoculum was streaked on the surface of Mueller–Hinton agar (Merck) using a sterile cotton swap to form an even lawn. Then, sterile paper disc (6 mm diameter) impregnated with 20 μl of 10 mg/ml EoNP was placed on the inoculated agar. Then, 20 μl of 40 μg/ml chloramphenicol was used as drug control and 20 μl of 10 mg/ml ChNP as negative control. All plates were incubated at 37 °C for 24 h. The diameters of the inhibition zone were then measured in millimetres after the incubation period. Three replicates of experiments were performed in separate occasions.

Besides, broth microdilution assay was performed to determine the minimal inhibitory concentration (MIC) and minimal lethality concentration (MLC) of the essential oil using sterile flat-bottom 96-well plate (NEST)^11^. Only test microorganisms that showed significant inhibitory activity on disc diffusion assay were tested. Checkerboard assay was performed to determine the interactions between two test substances^12^. The fractional inhibitory concentration (FIC) index was calculated based on the following equation: $\sum {\rm{FIC}}={{\rm{FIC}}}_{{\rm{A}}}+{{\rm{FIC}}}_{{\rm{B}}}=({{\rm{C}}}_{{\rm{A}}}/{{\rm{MIC}}}_{{\rm{A}}})+({{\rm{C}}}_{{\rm{B}}}/{{\rm{MIC}}}_{{\rm{B}}})$, where MIC_A_ and MIC_B_ are MICs of drug alone, whereas C_A_ and C_B_ are the concentrations of drug in combination. Synergistic effect is observed when $\sum {\rm{FIC}}\le 0.5$, indifferent when $\sum {\rm{FIC}} > 0.5 < 4.0$, and the cbination is defined as antagonistic when $\sum {\rm{FIC}}\ge 4.0$.

Kill curve study was performed to study the effect of EoNP ccentration on microbial growth^13^. EoNP was tested at four final concentrations: IC, 2 × MIC, MLC and 2 × MLC. Then, 50% methanol was included as negative control. Every 6 h, 500 μl of the samples was withdrawn and the viable cell count was determined by spread plate method. The kill curves were plotted using logarithm of viable cells against incubation time.

Acollagen model mimicking diabetic wound was adopted to study the efficacy of EoNP^14^. Bacteria cultures were prepared in 5 ml sterile tryptic soya broth (Merck) and incubated at 37 °C with rotational speed of 120 rpm in an incubator shaker. After incubating overnight, the bacteria cultures were diluted to 10^5^ cfu/ml in simulated wound fluid (SWF). SWF is a mixture of 50% fetal bovine serum (Merck) and 50% of peptone water, composed of 0.9% NaCl in 0.1% peptone (Merck). The colony counts of SWF in insert and well were determined using viable cell count on Mueller–Hinton agar.

## Results

**Figure 1:**
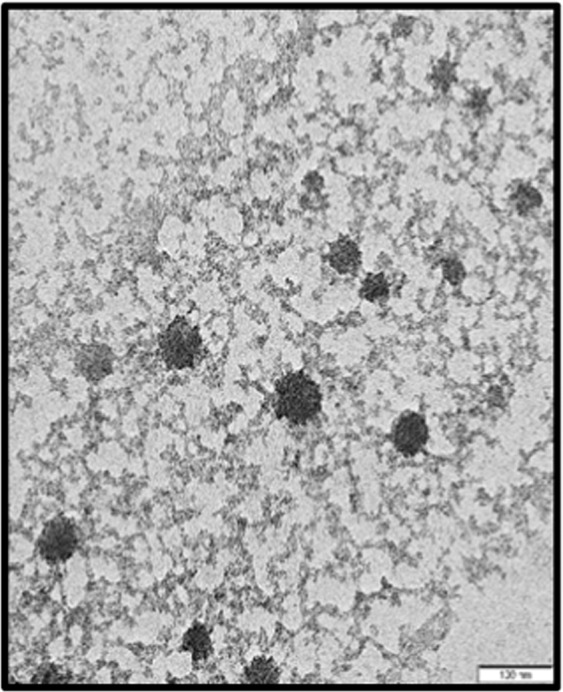
TEM images of the nanoparticles morphology and average size of EoNP.

**Figure 2:**
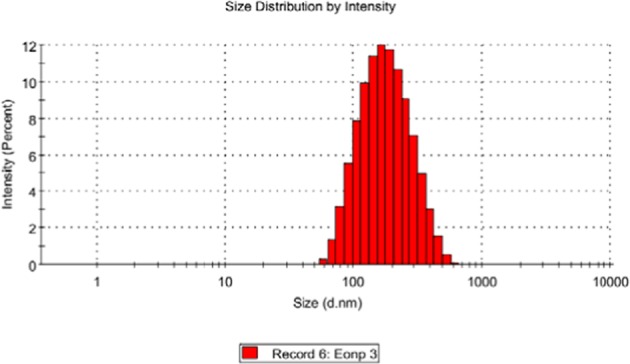
Size distribution by intensity for aqueous *H*. *pineodora* essential oil loaded chitosan nanoparticles.

The nanoparticles of the essential oil were successfully synthesised. The particles showed an average size of 70 ± 20 nm with spherical in shape based on microscopic observation of the TEM micrograph in Fig. 1. EoNP exhibited a small mean particles size that is less than 100 nm. However, the Zetasizer analysis shows the particle size distribution for EoNP with diameter of 158.70 nm (Fig. 2). Polydispersity index of particles was 0.176. The particles also showed a positive surface charge, with a zeta potential value of +24.10 mV. A similar range of zeta potential for ChNPs was reported^15^.

**Figure 3:**
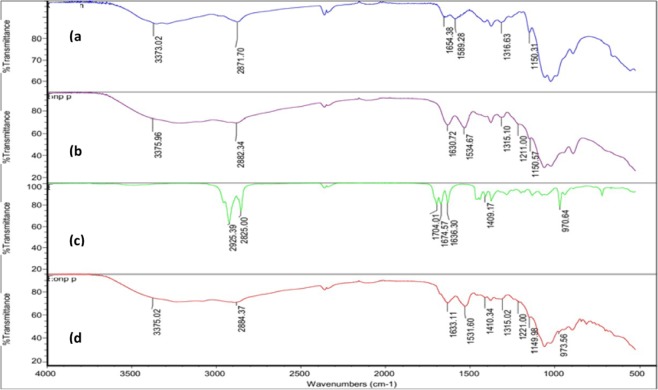
FTIR spectra of (**a**) pure chitosan, (**b**) chitosan nanoparticles, (**c**) H. pineodora essential oil and d) H. pineodora essential oil nanoparticles.

Absorption in the infrared region is due to the changes in the vibrational energy within the molecules (Fig. 3). In general, characteristic absorptions of chitosan were observed at 3000–3500 cm^−1^ (–OH, NH_2_ stretching), 2871 cm^−1^ (C–H stretching), 1654 cm^−1^ (C–N stretching of amide I), 1589 cm^−1^ (C–N stretching of amide II), 1316 cm^−1^ (C–N stretching of amide III) and 1150 cm^−1^ (C–O–C stretching)^16^. For ChNP, the stretching of amide I and amide II was shifted to 1630 and 1534 cm^−1^, respectively. The absorption at 1211 cm^−1^ was attributed to P = O, which crosslinked between the phosphoric groups of TPP and ammonium ions of the chitosan within the nanoparticles^17^. For Eo, several bands were observed at 2925 cm^−1^, 2852 cm^−1^ (C–H stretching), 1704 cm^−1^ (C = O stretching of ketone), 1636 cm^−1^ (C = C stretching of alkene), 1409 cm^−1^ (O–H bending) and 970 cm^−1^ (C–H bending), indicating a complex mixture of compounds present.

**Figure 4:**
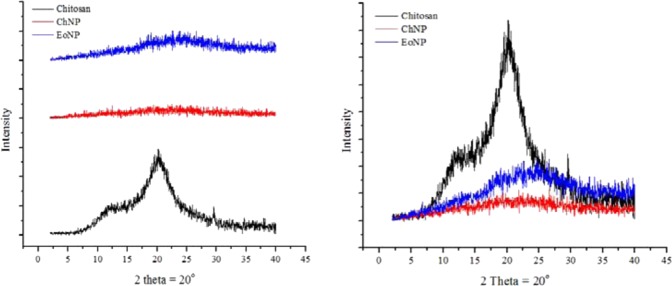
XRD patterns of chitosan, chitosan nanoparticles and *H*. *pineodora* essential oil loaded chitosan nanoparticles.

The crystal structures of chitosan, ChNP and EoNP and the XRD were elucidated (Fig. 4). A typical peak of chitosan was observed at 2θ = 12°. Another broaden peak of chitosan was noticed at 2θ = 20°, which indicates a higher crystallinity of the chitosan structure. The data were similar to that in the previous study^18^. It is observed that both peaks were not found after crosslinking with TPP during formation of ChNPs, reflecting the destruction of native chitosan structure^9^.

**Figure 5:**
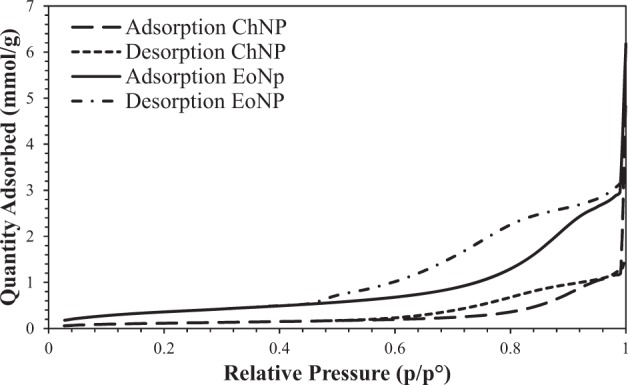
BET nitrogen adsorption–desorption isotherm of ChNP and EoNP.

Both BET plots of EoNP and ChNP were identified as type IV nitrogen adsorption–desorption isotherm, which is described to present a cylindrical pores and channel-like pore structures (Fig. 5). This isotherm type showed typical type IV characteristics, which indicates that the average pore sizes were larger than micro-pores. Both EoNP and ChNP showed typical H3 hysteresis loop that can be seen at 0.45 > p/p° > 0.9 and 0.6 > p/p° > 0.9, respectively. This loop is commonly associated with capillary condensation of nitrogen gas inside the pores at high relative pressure^19^. These capillary condensation steps revealed that both EoNP and ChNP consist of slit-shaped pores. The results showed that EoNP showed broader loop than ChNP. This indicates a large number of slit-shaped pores present in EoNP (Table 1). The surface area of EoNP (31.15 m^2^/g) was higher than ChNP (9.73 m^2^/g) because of the high number of pores that exist. Furthermore, EoNP has a larger volume (0.15 cm^3^/g) than ChNP (0.09 cm^3^/g). The higher adsorption would be expected because of the pores that exist.

**Table 1:** The surface characteristics analysis on ChNP and EoNP based on BET analysis.

Sample	Surface area (m^2^/g)	Total pore volume (cm^3^/g)	Average pore diameter (nm)	Average pore width (nm)
ChNP	9.73	0.09	38.79	16.77
EoNP	31.15	0.15	19.71	11.61

- = no inhibition zone.

**Figure 6:**
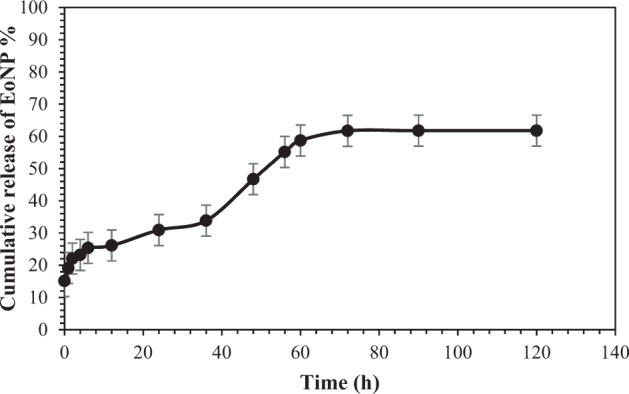
*In vitro* release profiles of *H*. *pineodora* essential oil from chitosan nanoparticles prepared in phosphate buffer saline pH 5.5.

The EE and LC obtained were 27.56% and 25.60%, respectively. It is relatively higher than that in the previous study^9^. For the drug release study, the release reached plateau after 72 h (Fig. 6). The total release of EoNP was 61.8% ± 0.08. Two burst release patterns were observed in the EoNP release profile. For the first 12 h, the initial burst release effect was observed with 25.39 ± 0.05% of drug released followed by slow release phase and another burst release effect at 60 h.

On disc diffusion assay, 8 out of 14 test microorganisms were inhibited by *H*. *pineodora* essential oil with diameter of clear zone ranged from 7.0 to 11.0 mm (Table 2). No inhibition zone was observed for controls, which indicates that the inhibitory activity was solely due to the presence of *H*. *pineodora* essential oil. EoNP displayed antimicrobial activity against the test microorganisms, with an MIC range of 4.88–78.00 μg/ml (Table 3). The broad spectrum of activities could be due to the synergistic effect of the essential oil with ChNP. A higher MLC was obtained for *E*. *coli*, *K*. *pneumoniae*, *A. anitratus*, *S. typhimurium* and *P*. *aeruginosa* (625.00 μg/ml). The lowest MIC was obtained on *B. cereus*, *Yersinia* sp., *E. coli*, *A. anitratus* and *S. typhimurium* (4.88 μg/ml).

**Table 2:** Antimicrobial activities of *H. pineodora* essential oil, EoNP and ChNP on disc diffusion assay tested on various diabetic wound pathogens.

Test microorganisms	Diameter of inhibition zone (mm)				
	Essential oil	Essential oil nanoparticles (EoNP)	Chitosan nanoparticles (ChNP)	Chloramphenicol	Negative control (50% methanol)
**Gram-positive bacteria**					
*S. aureus*	9.0 ± 0.1	37.0 ± 0.1	7.0 ± 0.1	11.0 ± 0.1	—
*B. cereus*	10.7 ± 0.2	36.0 ± 0.1	7.0 ± 0.1	11.0 ± 0.1	—
*B. subtilis*	11.0 ± 0.1	31.0 ± 0.1	—	13.0 ± 0.1	—
MRSA	9.0 ± 0.1	32.0 ± 0.1	—	11.0 ± 0.1	—
**Gram-negative bacteria**					
*P. mirabilis*	9.3 ± 0.1	38.0 ± 0.1	15.0 ± 0.1	13.0 ± 0.1	—
*Yersinia* sp.	9.0 ± 0.1	46.0 ± 0.1	10.0 ± 0.1	15.0 ± 0.1	—
*E. coli*	9.0 ± 0.1	34.0 ± 0.1	8.0 ± 0.1	11.0 ± 0.1	—
*K. pneumoniae*	—	38.0 ± 0.1	—	10.0 ± 0.1	—
*A. anitratus*	10.0 ± 0.1	36.0 ± 0.1	7.0 ± 0.1	13.0 ± 0.1	
*S. boydii*	—	31.0 ± 0.1	7.0 ± 0.1	13.0 ± 0.1	—
*S. typhimurium*	—	38.0 ± 0.1	7.0 ± 0.1	11.0 ± 0.1	—
*P*. *aeruginosa*	9.0 ± 0.1	32.0 ± 0.1	7.0 ± 0.1	11.0 ± 0.1	—
**Yeasts**					
*C. utilis*	—	28.0 ± 0.1	—	10.0 ± 0.1	—
*C. albicans*	10.0 ± 0.1	11.0 ± 0.1	8.0 ± 0.1	12.0 ± 0.1	—

**Table 3:** The susceptibility of test microorganisms to *H. pineodora* essential oil loaded-chitosan nanoparticles on broth microdilution assay.

Test microorganisms	MIC (μg/ml)	MLC (μg/ml)
**Gram-positive bacteria**		
*S. aureus*	9.75	39.00
*B. cereus*	4.88	19.50
*B. subtilis*	9.75	39.00
MRSA	9.75	39.00
**Gram-negative bacteria**		
*P. mirabilis*	19.50	156.25
*Yersinia* sp.	4.88	156.25
*E. coli*	4.88	625.00
*K. pneumoniae*	9.75	625.00
*anitratus*	4.88	625.00
*S. boydii*	9.75	39.00
*S. typhimurium*	4.88	625.00
*P*. *aeruginosa*	78.00	625.50
**Yeasts**		
*C. utilis*	9.75	39.00
*C. albicans*	39.00	312.50

The synergistic effect of *H*. *pineodora* essential oil in conjugation with biologically synthesised ChNPs was evident in the FIC indices shown in Table 4. Overall, no indifferent and antagonistic FIC index (≥4.0) was observed in this study for all test microorganisms. All test microorganisms showed a low FIC index (≤0.5) on checkerboard assay, which indicates a synergism effect (90.0–99.9%) between the combination of ChNP and *H*. *pineodora* essential oil.

**Table 4:** Fractional inhibitory concentration (FIC) indices of ChNP and *H. pineodora* essential oil using checkerboard assay.

Test microorganisms	*H*. *pineodora* essential oil	Chitosan nanoparticles (ChNP)	Combination (EoNP)	FIC index
	MIC_A_ (μg/ml)	MIC_B_ (μg/ml)	C_A_C_B_ (μg/ml)	
**Gram-positive bacteria**				
*S. aureus*	1250.0	156.25	9.75	0.0702, synergism
*B. cereus*	312.50	156.25	4.88	0.0468, synergism
*B. subtilis*	156.25	10000.00	9.75	0.0634, synergism
MRSA	156.25	10000.00	9.75	0.064, synergism
**Gram-negative bacteria**				
*P. mirabilis*	312.50	78.00	19.50	0.3124, synergism
*A. anitratus*	312.50	156.25	4.88	0.0468, synergism
*S. boydii*	312.50	156.25	9.75	0.0936, synergism
**Yeast**				
*C. albican*	625.00	156.25	39.00	0.039, synergism

$$\sum {\rm{FIC}}\le 0.5={\rm{synergism}}$$.

$$\sum {\rm{FIC}} > 0.5 < 4.0={\rm{indifferent}}$$.

$$\sum {\rm{FIC}}\,\ge 4.0={\rm{antagonism}}$$.

**Figure 7:**
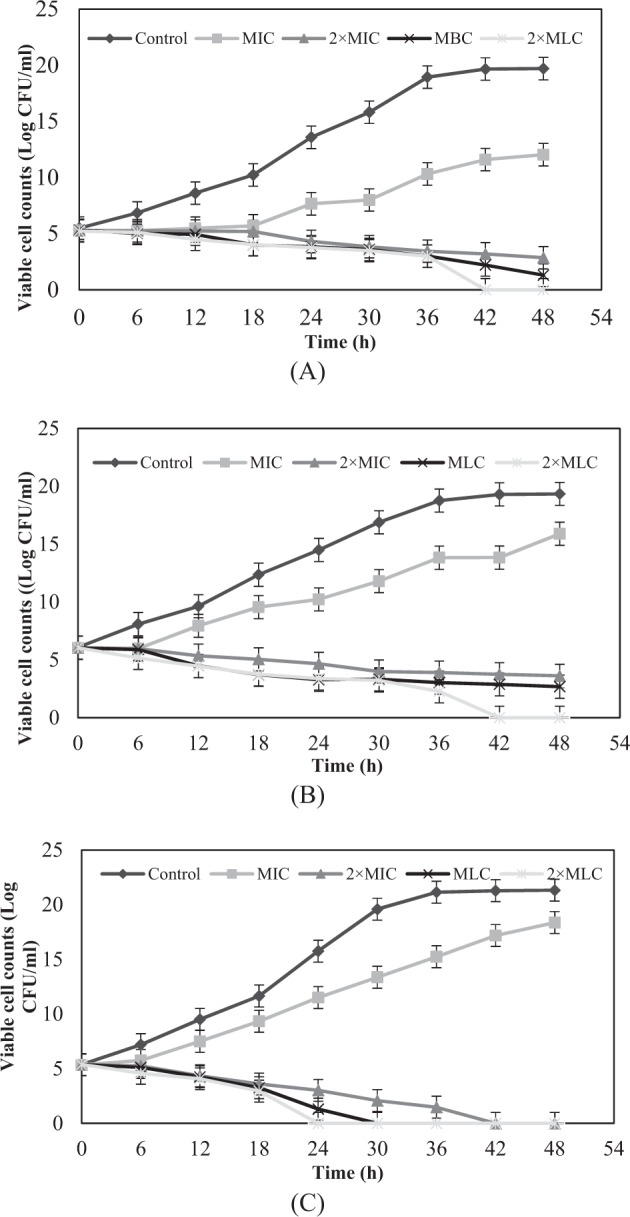
Growth curves of (**A**) *S. aureus*, (**B**) *P*. *aeruginosa* and (**C**) *C. utilis* exposed to four different concentrations of EoNP for a duration of 48 h.

Figure 7 shows a typical pattern of kill curves on *S. aureus*,. *aeruginosa* and *C. utilis* at different concentrations of EoNP. A lag phase was observed in *S. aureus* for the first 6 h with lower viable cell counts. All growth control curves exhibited four distinct phases, namely, lag phase, log phase, stationary phase and death phase. The growth of *S. aureus* decreased gradually at both 2 × MIC and MLC. This bacterium showed 99.9% of growth reduction at 2 × MLC after 42 h of exposure. No log phase was observed in the kill curve. The viability of *P*. *aeruginosa* was eradicated within 42 h at 2 × MLC, similar to *S. aureus*. However, the viable cell count of *P*. *aeruginosa* was higher than of *S. aureus* at MIC. The rapid increment of viable cell count was also observed in *C. utilis* at the same concentration.

A new collagen wound model established that mimics the 1B diabetic foot ulcer was adopted to test the antimicrobial efficiency of EoNP in an artificial wound model^14^. The percentage reduction of viable cell count for all test microorganisms was ranged from 61.77% to 83.03% in collagen and from 61.07% to 80.71% in exudate (Table 5). The result was variable as each test microorganisms may have different modes of growth.

**Table 5:** Percentage of growth reduction exhibited by EoNP on 3D collagen wound model.

st microorganisms	Percentage of growth reduion (%)	
	Collagen	Exudate
**Gram-positive bacteria**		
*S. aureus*	76.00 ± 7.4	69.35
*B. cereus*	83.03 ± 7.4	72.88
*B. subtilis*	73.64 ± 7.4	67.26
MRSA	73.66 ± 7.4	60.39
**Gram-negative bacteria**		
*P. mirabilis*	61.8 ± 5.4	62.2 ± 6.2
*Yersinia* sp.	65.9 ± 5.1	61.1 ± 7.9
*E. coli*	71.9 ± 4.8	63.3 ± 3.4
*K. pneumoniae*	77.7 ± 6.1	79.6 ± 3.1
*A. anitratus*	69.8 ± 4.8	65.3 ± 4.9
*S. boydii*	79.4 ± 2.8	80.7 ± 5.2
*S. typhimurium*	82.1 ± 4.4	71.3 ± 3.6
*P*. *aeruginosa*	72.3 ± 2.8	67.2 ± 3.1
**Yeasts**		
*C. utilis*	75.28 ± 7.4	79.4 ± 2.7
*C. albicans*	73.64 ± 6.4	67.3 ± 6.2

## Discussion

EoNPs were prepared using ionic gelation method. Chitosan was selected to encapsulate EoNP because of its biodegradability, cationic charge and muco-adhesive characteristic^15^. Nanoparticles prepared using ionic gelation between negatively charged TPP and positively charge chitosan has been widely used in drug delivery studies as an encapsulant for essential oil^9^. The average size obtained from TEM micrograph was similar to that in the previous study that demonstrated a normally distributed size of cardamom essential oil in ChNPs with a range of 75 ± 25 nm^11^. A similar range of diameter has also been previously reported using oil-in-water emulsion method and Tween 80 as emulsifier^20^. The size reported previously was in the wide range of 40–500 nm, which mainly depends on the synthesis method^9^. The size of particles tested using DLS was significantly larger compared with TEM images. This is possibly due to the agglomeration of nanoparticles in the liquid medium. This result was supported by previous reports^9,21^.

The surface chemistry of nanoparticles affects the interaction with the cells in physiological environment during drug delivery. Besides, other factors like size and shape of particles also play a key role on the performance of the nanoparticles^22^. When interacting with TPP, the excess positive charge of chitosan contributes the strong positive charge^9^. In general, the EoNPs are thermally stable due to the large electrostatic repulsion energy between particles trace. Thus, the likelihood to aggregate is very low^23^. Electrostatic force between positively charged particles promotes a closer interaction with negatively charged bacteria leading to the penetration of the drug through bacteria cell wall^9^. So, the potential of the nanoparticles to gather at the infection site will increase. Based on literature, the positively charged particles were able to alter the electron transport chain of bacterial membrane^11^.

The FTIR spectrum showed that when essential oil was loaded in ChNP, most of the peaks on essential oil disappeared. In addition, the absence of new absorptions in the EoNP suggested that essential oil and ChNP are physically bound to each other without any chemical interactions and modifications. Besides, the decreasing crystallinity of chitosan on XRD pattern is partially due to the ChNPs that consist of a dense network structure of polymer chains of chitosan crosslinking by TPP counter-ions^24^. Thus, there is existence of an amorphous structure in ChNPs. Intensities of peak were increased as the ChNPs were loaded with *H*. *pineodora* essential oil. This implies the change of structure when incorporation with TPP and chitosan^25^. Based on BET results, a reduction of pore size of nanoparticles from 38.79 to 19.71 nm was observed after loading the *H*. *pineodora* essential oil. The results suggest that *H*. *pineodora* essential oil fills the pores that existed in the ChNP matrix.

A relatively high encapsulation efficiency and loading capacity were recorded in this study. This is contributed by the optimal stirring during the preparation of the nanoparticles. In general, a high-concentration essential oil also reduces the EE^26^. This is due to the saturation of *H*. *pineodora* essential oil in the polymer matrix. Thus, to increase the EE and LC, a lower weight ratio of chitosan to essential oil should be used.

The *in vitro* release profile of essential oil from ChNPs was studied using phosphate buffer saline as medium at pH 5.5 as stage I ulcer has an acidic pH (pH 5.4–5.6)^9^. The initial burst release effect was possibly due to the essential oil molecules present on the surface of polymer by adsorption^17^. This is necessary to provide an appropriate initial amount of essential oil to combat the pathogens on wound. The drug release at later phase was sustained more than 24 h as the essential oil gradually dispersed from the nanoparticles. At this stage, the polymer matrix started to swell because of the penetration of the buffer solution. The second burst release pattern occurred at 36 h. The release of the essential oil was complete on Day 3 following the first order of kinetic. The release of a drug through the skin depends on the physicochemical properties of the drug itself combined with the influence of the vehicle to alter the drug penetration profile. This study suggests that the release pattern is suitable for wound system because it can reduce the increasing frequency of the dressing.

On disc diffusion assay, all test substances were more susceptible to Gram-positive bacteria than to Gram-negative bacteria. This is in agreement with the other study that Gram-positive bacteria were more sensitive to essential oil because of the presence of lipopolysaccharides in the cell wall^27^. In general, EoNP demonstrated significant inhibitory activity against all test microorganisms. An inhibition zone (*p* ≤ 0.05) was observed to be significantly larger compared with essential oil for all test microorganisms. The antimicrobial activity of the ChNP was compared with EoNP. However, the diameter of the clear zone was significantly smaller (*p* ≤ 0.05). Among all the test microorganisms, *Yersinia* sp. showed the highest susceptibility that is evident in the large inhibition zone of 46.0 mm. In addition, EoNP also showed antifungal activity against yeasts with a smaller zone size compared with test bacteria. The MLC results were significantly higher than MIC results aa higher EoNP concentration was needed to kill the test microorganisms. The antimicrobial activity of EoNP was concentration-dependent. *P*. *aeruginosa* was the most resistant to EoNP based on the MIC. This bacterium also showed small inhibition zone in disc diffusion assay.

Ocheckerboard assay, against all test microorganisms, we noticed that the combination treatment of *H*. *pineodora* essential oil and ChNP resulted to a lower MIC compared with essential oil alone. The results in this study showed that the combination of ChNP and Eo decreased the MICs as much as on all test microorganisms. Therefore, the combination of both ChNP and *H*. *pineodora* essential oil increased the susceptibility of the test microorganisms. Kill curves are used to study the pharmacokinetics of an antimicrobial agent. The results showed that the viable cell counts were significantly reduced with the increasing of EoNP concentration. The antimicrobial efficacy of EoNP was concentration-dependent. At the MIC, 99.9% of killing was not achieved for all test microorganisms. A gradual reduction in viable cell count at MLC and 2 × MLC were shown on all test microorganisms. The data were in agreement with the susceptibility assay. At all EoNP concentrations, no post-antibiotic effect was observed for all test microorganisms in this study.

A grade 1B ulcer was classified as an infected superficial wound that does not include tendon, capsule or bone^28^. This model utilised type I collagen because this type of collagen is most abundant in the dermis^29^. Following the biofilm formation, the presence of type I collagen also was identified as an effective substrate for bacterial attachment^30^. Therefore, the characteristic is closer to the *in vivo* situation. The combination of collagen matrix and biofilm may limit the oxygen intake to the biofilm that may contribute to robust biofilm formation compared with microdilution assay^14^. This study suggests that the *H*. *pineodora* essential oil was not fully released from ChNP after 24 h. This is in agreement with the results of drug release study that demonstrated that the essential oil can be fully released from EoNP after 72 h. Therefore, 99.9% of growth reduction was not obtained on all test strains in this study. To conclude, the synthesis of EoNP significantly enhanced the antimicrobial activity of *H*. *pineodora* essential oil. The antimicrobial synergistic effect between *H*. *pineodora* essential oil and ChNP was evident in the low FIC index on checkerboard assay.

In conclusion, the nanoparticles of *H*. *pineodora* essential oil were successfully developed. The encapsulation of *H. pineodora* essential oil into chitosan nanoparticles was validated FTIR spectra, XRD analysis and BET studies. The release of essential oil can be prolonged to 3 days, which is ideal for diabetic wound care. The synthesis of EoNP significantly enhanced the antimicrobial activity of *H. pineodora* essential oil. The antimicrobial synergistic effect between *H. pineodora* essential oil and ChNP was evident by low FIC index on checkerboard assay. A 3D collagen wound model was successfully developed in this study to test the antimicrobial efficacy of the nanoparticles. The essential oil nanoparticles significantly reduced the bacterial burden of the wound, on both collagen and exudate. To test the *in vivo* efficacy of the synthesised nanoparticles, further work should be extended. Investigations should be carried out to design a wound dressing material incorporated with *H. pineodora* essential oil nanoparticles to provide a therapeutic alternative to combat microbial infections on chronic wounds.
